# A Novel T55A Variant of G_s_ *α* Associated with Impaired cAMP Production, Bone Fragility, and Osteolysis

**DOI:** 10.1155/2016/2691385

**Published:** 2016-08-07

**Authors:** Kelly Wentworth, Alyssa Hsing, Ashley Urrutia, Yan Zhu, Andrew E. Horvai, Murat Bastepe, Edward C. Hsiao

**Affiliations:** ^1^Division of Endocrinology, Diabetes, and Metabolism and The Institute for Human Genetics, Department of Medicine, University of California, San Francisco, San Francisco, CA 94143, USA; ^2^Endocrine Unit, Massachusetts General Hospital and Harvard Medical School, Boston, MA 02114, USA; ^3^Departments of Pathology and Laboratory Medicine, University of California, San Francisco, San Francisco, CA 94143, USA

## Abstract

G-protein coupled receptors (GPCRs) mediate a wide spectrum of biological activities. The GNAS complex locus encodes the stimulatory alpha subunit of the guanine nucleotide binding protein (G_s_α) and regulates production of the second messenger cyclic AMP (cAMP). Loss-of-function GNAS mutations classically lead to Albright's Hereditary Osteodystrophy (AHO) and pseudohypoparathyroidism, often with significant effects on bone formation and mineral metabolism. We present the case of a child who exhibits clinical features of osteolysis, multiple childhood fractures, and neonatal SIADH. Exome sequencing revealed a novel* de novo* heterozygous missense mutation of GNAS (c.163A<G, p.T55A) affecting the p-loop of the catalytic G_s_α GTPase domain. In order to further assess whether this unique mutation resulted in a gain or loss of function of G_s_α, we introduced the mutation into a rat GNAS plasmid and performed functional studies to assess the level of cAMP activity associated with this mutation. We identified a 64% decrease in isoproterenol-induced cAMP production* in vitro*, compared to wild type, consistent with loss of G_s_α activity. Despite a significant decrease in isoproterenol-induced cAMP production* in vitro*, this mutation did not produce a classical AHO phenotype in our patient; however, it may account for her presentation with childhood fractures and osteolysis.

## 1. Introduction

Mutations affecting the GNAS complex locus can lead to either activation or inhibition of G_s_ *α* [1, 2]. Several diseases result from GNAS mutations, including those that activate G_s_ *α* (McCune-Albright Syndrome and fibrous dysplasia of the bone) [3] and those that inhibit G_s_ *α*, such as AHO and pseudohypoparathyroidism [1, 4–7].

GNAS is a highly complex locus encoding multiple products with exclusively maternal or paternal expression. While G_s_ *α* expression is biallelic in most tissues, the paternal G_s_ *α* promoter is silenced in the proximal renal tubules, pituitary, thyroid, and gonads. Consequently, the phenotype of patients with inactivating GNAS mutations differs depending on which parental allele is affected. Patients with AHO exhibit characteristic features including obesity, brachydactyly, shortened fourth metacarpals, short stature, subcutaneous ossification, and occasionally cognitive impairment [1]. AHO has two common subtypes: pseudohypoparathyroidism (PHP-Type-Ia) and pseudopseudohypoparathyroidism (PPHP). PHP-Type-Ia occurs when the maternal allele harbors the mutation and causes classical AHO features and end-organ resistance to PTH in the proximal tubule. These patients often show resistance to growth-hormone-releasing hormone, thyroid-stimulating hormone, and gonadotropins. In contrast, when the paternal GNAS allele is affected, patients exhibit AHO features without PTH resistance (PPHP), since the paternal allele is silenced in the proximal tubule. This is distinct from patients with activating GNAS mutations as seen with McCune-Albright Syndrome (MAS), who typically present with the classic triad of polyostotic fibrous dysplasia of the bone, café-au-lait skin hyperpigmentation, and precocious puberty. MAS is also a mosaic disease, and, depending on the degree of mosaicism, patients can present with other endocrinopathies including hyperthyroidism, acromegaly, and Cushing's syndrome. Here, we present a young female with osteolysis, multiple fractures, and a history of SIADH, who harbors a novel inactivating GNAS mutation.

## 2. Case Presentation

Our patient was born to nonconsanguineous parents of Asian descent. Pregnancy was complicated by oligohydramnios and prematurity at 34-3/7 weeks. She developed respiratory distress syndrome requiring intubation for the first 24 hours. She was severely hyponatremic (Na^+^ = 116 mmol/L) and did not respond to sodium chloride or glucocorticoids. No hypoaldosteronism or ADH receptor gene mutations were found. She was diagnosed with neonatal SIADH and successfully treated with fluid restriction. She also had a congenital fracture of her right femoral diaphysis. A bone survey at two days of age showed skeletal hypomineralization.

**Figure 1:**
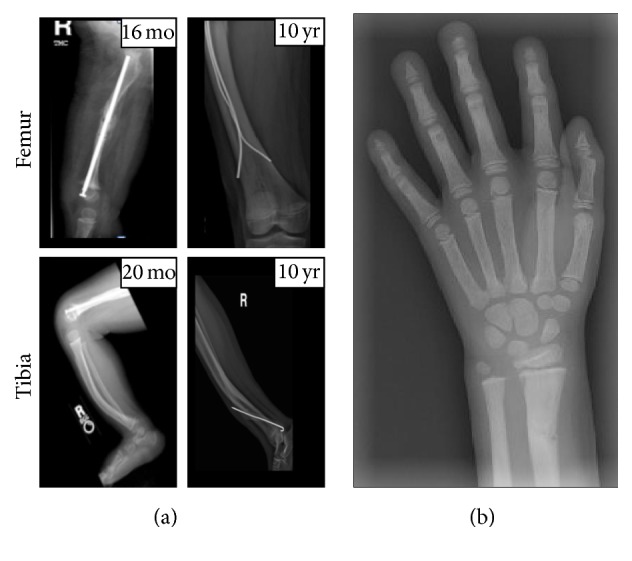
(a) Right femoral diaphysis rod and bowing of the tibia at 16 months of age, 20 months, and 10 years. (b) Arrow depicting acroosteolysis of the L hand (age: 6).

During early childhood, she developed bowing of her femora, tibiae, and fibulae and a two-centimeter leg length discrepancy. At age 16 months, she sustained a closed fragility fracture of her right femoral diaphysis requiring fixation with a Bailey-Dubow rod (Figure 1). A bone survey at 16 months of age demonstrated possible acro-osteolysis with a metaphyseal corner fracture of her right tibia. At 20 months, a repeat bone survey showed osteolysis of the distal phalanges, metacarpals, and metatarsals and bowing of the femora, tibiae, and fibulae.

She was evaluated by a medical geneticist who felt her clinical picture was consistent with an unspecified osteolytic syndrome. A bone biopsy showed increased bone resorption and mild hypomineralization without collagen defects. She received IV pamidronate from ages two to four for fracture prevention with a subsequent *Z*-score of −1.1 on DEXA and stabilization of osteolysis. At the age of four, she was transitioned to 35 mg of oral alendronate weekly but sustained right fibular and bilateral wrist fractures in the setting of minimal trauma. At the age of six, the alendronate was increased to 70 mg weekly. This was discontinued one year later when she had achieved a normal BMD for her age (*Z*-score: +0.4).

**Figure 2:**
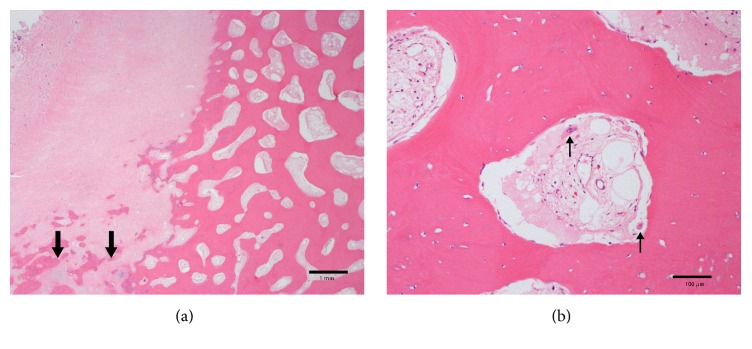
(a) The R fibula pseudarthrosis biopsy from 2011 demonstrated a fibroosseous lesion (top left) contiguous with fracture callus-type changes (arrows) buttressed on lamellar bone (right); H&E stain; scale bar: 1 mm. (b) Bone resorption of native bone was not increased as osteoclasts were inconspicuous and uncommon (arrows); H&E stain; scale bar: 100 microns.

At the age of seven, she developed right ankle pain and X-ray imaging confirmed pseudarthrosis of her distal fibula. She underwent excision, bone grafting, and intramedullary nail placement. Bone pathology showed pseudarthrosis of the right fibula and presence of cortical-type bone without increased turnover or resorption (estimated osteoclast activity <5%). We could not assess the degree of hypomineralization due to the decalcification used to process and section the bone tissue (Figure 2). At the age of nine, she sustained another right femoral fracture requiring intramedullary nail placement, which was complicated by delayed healing. Intraoperative bone biopsy demonstrated fibroosseous proliferation of the lesion and scant normal-appearing bone, thought to be related to the intramedullary nail. Again, hypomineralization could not be assessed as the samples were decalcified. A subsequent DEXA confirmed stable BMD (*Z*-score: +0.5). At the age of 10, she refractured her fibula around the hardware.

In addition to her bony abnormalities, she was diagnosed with probable Asperger's syndrome and attention deficit hyperactivity disorder. She underwent menarche at the age of nine. Due to concern for possible precocious puberty, she was evaluated by a pediatric endocrinologist, who determined that the age of nine was consistent with normal menarche. She otherwise developed normally. Her prior diagnosis of SIADH has been managed successfully with fluid restriction.

**Figure 3:**
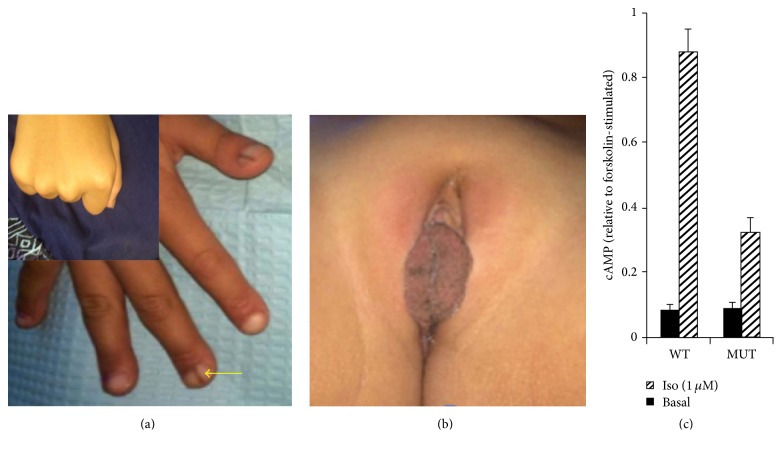
(a) Shortened distal phalanges with arrow depicting Bonde's lines. Insert shows no evidence of shortened fourth metacarpal. (b) Labia minora hyperplasia without clitoromegaly. (c) G_s_ *α*-null mouse embryonic fibroblasts transfected with cDNA encoding the c.163A>G mutant showed a 64% reduction in isoproterenol-induced cAMP production compared to WT G_s_ *α*. The difference between the WT and mutant isoproterenol-induced cAMP accumulations is statistically significant (*p* < 0.01).

Physical examination revealed normal vital signs and no abnormal facies except bilateral iris hypopigmentation. She has four small (0.5–1.2 cm) hyperpigmented spots on her right flank and multiple keloids from orthopedic procedures. She has mild bowing of both legs without tenderness, bony clubbing of her fingertips, shortened distal phalanges, and hyperpigmented nail lines. She has labia minora hyperplasia and hyperpigmentation without clitoromegaly (Figure 3). The remainder of her examination was normal. Notably, she does not have the short stature, shortened fourth metacarpals, obesity, or subcutaneous ossifications classically seen in AHO. Her laboratory evaluation, including calcium, PTH, and vitamin D, was unremarkable except for mild hyperphosphatemia (Table 1).

Comparative genomic hybridization (CGH) array analysis showed no deleterious deletions or duplications in either the patient or her parents. The CGH was performed through Sunquest Laboratories (#M14494; SignatureChipOS v2 12-plex; 134829 Oligo Probes) on a DNA sample from the patient's blood. The microarray covered 3397 loci across the whole genome including subtelomeric regions and pericentromeric regions. No clinically significant abnormalities were identified in these studies. Since microarray analysis could miss a small deletion and is insensitive for sequence variants, the patient subsequently underwent whole exome sequencing (Ambry Genetics) from lymphocyte DNA which revealed a* de novo*, heterozygous missense mutation within GNAS exon 2 affecting the G_s_ *α* transcript (c.163A>G, p.T55A). The sequence read depth was 7x. Thirty additional gene variants were identified in addition to the GNAS c.163A>G variant (Supplemental Table  1 in Supplementary Material available online at http://dx.doi.org/10.1155/2016/2691385). Two were characterized as autosomal dominant variants that did not have any phenotypic correlation to our patient. The remaining variants were novel autosomal dominant and autosomal recessive variants with minor phenotypic overlap and were felt to be unlikely to produce clinically significant findings. Cosegregation analysis revealed that the unaffected parents do not carry the GNAS c.163G>A alteration, indicating that this likely represents a* de novo* mutation. No additional high-probability pathogenic variants were identified.

The GNAS c.163A>G missense mutation has not been reported previously in healthy cohorts. This mutation was not observed among any of the 6,502 individuals in the NHLBI Exome Sequencing Project. There are no data available for the allele frequency of this nucleotide change in the 1000 Genomes Project, and the mutation is not listed in the Database of Single Nucleotide Polymorphisms (dbSNP). This suggests that the GNAS c.163A>G mutation is exceedingly rare in healthy populations. The patient is of mixed heritage (Asian descent) and there is no data on the rate of this mutation in an ethnically matched population. A thorough literature search at the time of the original diagnosis did not identify any other patients who harbor this mutation.

## 3. cAMP Assay Methods and Results

This study was performed in accordance with the UCSF Committee on Human Research. A rat G_s_ *α*-HA cDNA plasmid [12] was modified using QuikChange mutagenesis (Invitrogen) with primers AU058 5′ GTCTGGCAAAAGCGCCATTGTGAAGCAG 3′ and AU059 5′ CTGCTTCACAATGGCGCTTTTCCAGAC 3′. Wild-type (WT) and mutant G_s_ *α*-HA plasmids were cotransfected with GloSensor plasmid (Promega, Madison, WI) using Lipofectamine® LTX with Plus*™* Reagent (Life Technologies, Grand Island, NY) into 2B2 cells [12] grown adherently in high glucose DMEM (SH30022.01, HyClone, Logan, UT, USA), 10% FBS (HyClone, South Logan, UT), and 1% penicillin/streptomycin (Corning Cellgro Mediatech, Manassas, VA) at 37°C in 5% CO_2_ and 95% O_2_ for cAMP analysis using an EnVision 2104 Multilabel Reader (PerkinElmer, Waltham, MA) [13]. The basal, unstimulated cAMP accumulation in the presence of 1 mM 3-isobutyl-1-methylxanthine (Sigma, St. Louis, MO) was comparable between cells expressing the WT or mutant G_s_ *α*. In contrast, the mutant G_s_ *α*-HA construct produced 64% less cAMP after isoproterenol stimulation compared to WT G_s_ *α*, when normalized to 10 *μ*M forskolin-stimulated cAMP levels (Figure 3).

## 4. Discussion

Our patient harbors a novel loss-of-function GNAS mutation and a phenotype of osteolysis, bone fragility, and fractures. Exome sequencing revealed a* de novo* missense mutation (c.163A>G, p.T55A) on exon 2 of GNAS. This mutation occurs within the p-loop of the GTPase domain of G_s_ *α* and may change its catalytic properties. We used the Ensemble Variant Effect Predictor model to determine if this variant might produce deleterious effects. The variant was considered “damaging” by SIFT, “probably damaging” by PolyPhen, and “deleterious” by both ConDel and Mutation Taster. Although this SNP was recently reported to be associated with MAS (rs797044895) [14], our data suggest otherwise since transfection of G_s_ *α*-null mouse embryonic fibroblasts with cDNA encoding the T55A mutant caused a 64% reduction in isoproterenol-induced cAMP production compared to wild-type G_s_ *α*. This strongly suggests that this mutation is associated with loss of G_s_ *α* activity. Interestingly, several other loss-of-function GNAS mutations that are associated with AHO also affect exon 2 (Table 2) [11]. This further supports our hypothesis that our patient's novel GNAS mutation lies in a biologically important region for G_s_ *α* activity.

Surprisingly, our patient lacks many classical features of AHO or PHP, such as obesity, subcutaneous calcifications, and major mineral ion metabolism abnormalities. This might reflect the partial loss of cAMP production from a hypomorphic allele. G_s_ *α* expression is typically biallelic in bone; thus, it is surprising that a 64% decrease in activity on one allele confers such dramatic bone fragility and osteolysis, given that we would anticipate 100% activity of the wild-type allele. We were unable to identify whether the GNAS mutation affected the maternal or paternal allele, since exome sequencing analysis does not have the ability to determine this. The absence of hormone resistance may suggest that the* de novo* somatic mutation occurred on the paternally inherited allele; however, we were unable to perform endocrine stimulation tests on the parents to exclude subclinical hormone resistance as the family was unavailable for detailed follow-up. An alternative possibility is that this mutation affects G_s_ *α* splicing, which could produce full loss of G_s_ *α* function. Furthermore, other GNAS transcripts utilize exon 2, and it is possible that our patient's phenotype reflects disrupted activity of another GNAS product, particularly one with monoallelic expression, such as XL*α*S. It is also possible that this amino acid substitution produces a dominant negative effect, accounting for the 64% decrease in cAMP accumulation. The mutation is in the alpha-1 helix and is facing out towards Arg201, a region considered to be within the p-loop. The mutation may weaken GTP binding since Thr55 is one of the 20 residues that are in contact with GTP and cause a loss of function through this mechanism. When we modeled the T55A protein using Swiss-Model, we confirmed that the GTP binding site was not conserved and did not fit the GTP into the ribbon model [15]. Finally, exome sequencing only detects mutations that occur in the coding portion of the genome; therefore, we cannot exclude the possibility that there are additional genetic mutations that occur in noncoding or regulatory sequences that could be contributing to this patient's phenotype. Further studies would be needed to elucidate these potential mechanisms.

We do not have a clear explanation for the prior history of hyponatremia; however, it is intriguing to hypothesize that there could be tissue-specific effects of this mutation because of the imprinting of the GNAS locus. There are 2 case reports in the literature describing 2 boys who harbor G_s_ *α* mutations (A366S) that caused both testotoxicosis and PHP. The A366S G_s_ *α* mutation constitutively activates cAMP* in vitro*, which explains the testotoxicosis, but the A366S protein is rapidly degraded at body temperature (37°C) resulting in functional loss of GNAS in other tissues and thus explaining the PHP phenotype. This is thought to be due to the lower temperature of the testes, which permits stability of the A366S G_s_ *α* mutation. A similar mechanism has been described with a G_s_ *α* mutation that led to neonatal diabetes due to hyperactivity but also caused PHP [16].

Our patient's comorbid diagnosis of early childhood SIADH suggests that this mutation could affect extraskeletal GPCR pathways, but the evaluation is confounded by the pulmonary complications at birth. While SIADH and bony abnormalities are not clinically linked, GPCRs are key mediators of both pathways. The mechanism of any link remains unclear, and the persistence of the SIADH remains unknown as she continues on self-imposed fluid restriction. How this GNAS mutation results in this pathology remains to be elucidated.

In summary, this case describes a patient with a unique phenotype of decreased BMD, osteolysis, and childhood fractures and identifies a novel exon 2 GNAS mutation which causes a decrease in cellular cAMP production.

## Supplementary Material

The supplementary material includes the additional thirty gene variants that were identified on exome sequencing.

## Figures and Tables

**Table 1:** Pertinent laboratory values.

Laboratory values	Birth	7 days^*∗*^	5 months^*∗*^	Age 10	Age 11	Age 11.5
Na^+^ (mmol/L)	116	131 (130–145)	120 (135–145)	137 (135–145)	137 (135–145)	—
Ca^++^ (mg/dL)	9.3	9.2 (8.0–11.5)	9.6 (8.0–11.5)	10.1 (8.8–10.3)	9.6 (8.8–10.3)	10.0 (8.8–10.3)
Phos (mg/dL)	5.8	5.0 (4.2–7.0)	5.8 (4.2–7.0)	5.7 (3.0–5.7)	6.8 (3.0–5.7)	4.9 (3.0–5.7)
Vit D 25-OH (ng/mL)	—	—	—	22 (30–100)	25 (30–100)	30 (30–100)
Serum osmolarity (mmol/kg)	—	281 (285–310)	262 (285–310)	299 (283–301)	293 (283–301)	—
Urine osmolarity	—	258	825 (300–1200 mosm/kg)	984 (300–900 mmol/kg)	947 (300–900 mmol/kg)	—
Urine cAMP (nmol/L)	—	—	—	7.4 (0.5–10.0)	—	—
PTH (ng/L)	27	—	—	—	57 (12–65)	20 (12–65)
Alkaline phosphatase	106 (<400 units/L)	—	—	—	—	92.7 (24.2–154.2 mcg/L)
Plasma renin activity (mg/mL/hr)	—	—	<20 (200–3500)	0.80 (0.25–5.82)	—	—
Aldosterone	—	29 (5–175)	4.4 (5–90)	—	—	—
Cortisol (mcg/dL) before and after stimulation	—	2.67 → 31.9 (4–26)	—	—	—	—
TSH	—	2.13 (mIU/mL)	—	—	—	0.76 (0.45–4.12 mIU/L)

Laboratory values at different points during childhood. *∗* indicates values obtained while receiving oral sodium chloride supplementation.

**Table 2:** Reported GNAS mutations in exon 2.

Nucleotide position	Protein position	Mutation type	Observed phenotype	Reference
c.144dupT	p.(Gly49Trpfs^*∗*^5)	Frameshift	AHO	[7]
c.150dupA	p.(Ser51IIefs^*∗*^3)	Frameshift	PHPIa, PPHP	[8]
c.188_189delTG	p.(Ser51IIefs^*∗*^3)	Frameshift	PHPIa, PPHP	[9]
c.191A>T	p.(His64Leu)	Missense	PHPIa	[10]
c.163A>G	p.(Thr55Ala)	Missense	Osteolysis, low BMD, frequent fractures	This case

Published GNAS mutations affecting exon 2. Adapted from Lemos and Thakker Human Mutation, 2015 [11].
